# The 1000 Canadian faces of systemic lupus erythematosus: effect of ethnicity on baseline pediatric data

**DOI:** 10.1186/1546-0096-10-S1-A18

**Published:** 2012-07-13

**Authors:** Deborah M Levy, Earl D Silverman, Lori B Tucker, Gaelle Chédeville, Adam Huber, Janet E Pope, Christine A Peschken

**Affiliations:** 1 BC Childrens Hospital, Vancouver, BC, Canada; 2 Hosp for Sick Children, Toronto, ON, Canada; 3 Hospital for Sick Children, Toronto, ON, Canada; 4 IWK Health Centre, Halifax, NS, Canada; 5 Montreal Children's Hospital, Montreal, QC, Canada; 6 St Joseph Health Care London, London, ON, Canada; 7 The Hospital for Sick Children, Toronto, ON, Canada; 8 University of Manitoba, Winnipeg, MB, Canada

## Purpose

To determine the influence of ethnicity and sociodemographic factors on disease presentation, manifestations, disease activity, and disease outcomes, using baseline data from the pediatric arm of the 1000 Canadian Faces of Lupus Study, a multicenter, prospective study of the Canadian Lupus population.

## Methods

Childhood-onset SLE (<18^th^ birthday) patients at four pediatric centers (Halifax, Montreal, Toronto and Vancouver). Collected data included sociodemographics, disease manifestations, current/past medications, laboratory measures and multiple disease measures. The Child Health Questionnaire (CHQ), measuring multiple domains of health status was administered. For analysis, patients were categorized by their primary self-selected ethnic category.

## Results

Between November 2005 and February 2009, 213 cSLE patients were enrolled. The number of patients enrolled at each site mirrored the size of the clinical centre: Toronto 134 (63%), Vancouver 54 (25%), Montreal 17 (8%), and Halifax 8 (4%). There were 176 (83%) females, mean age at diagnosis was 12.5 ± 0.3 years, mean disease duration was 2.5 ± 2.7 years, and 175 patients (82%) were born in Canada. Demographic data were similar across the geographic sites, except for a longer disease duration in Vancouver (3.9 ± 3.6 years, p<.001). Primary self-reported race/ethnicity data was available for 191 patients: White (31%), Asian (30%), South Asian (15%), Black (10%), Latino/Hispanic (4%), Aboriginal (4%) and Arab/Middle Eastern (3%). Because of low numbers, the Latino/Hispanic and Arab/Middle Eastern groups were excluded from the analysis. Ethnic distribution across the centers differed (p<.001), reflecting known differences in the urban populations. The distribution of household income, and prescription drug plan coverage did not differ across ethnicities, however, fewer Asians (64%) had dental insurance coverage as compared to White (88%) and Aboriginal (100%) patients (p<.01). Missed school days did not differ by ethnicity, although 26% of the entire cohort reported missing on average 6 days per month. CHQ scores were lower in 7 of 10 domains in white patients vs. non-white ethnicities (p<.05 for each). Autoantibodies and SLE classification criteria present at any time that differed by ethnicity are listed in table 1.

**Table 1:** Classification criteria by ethnicity

Criterion %present	Total (n=191)	Aboriginal (n=9)	Asian (n=63)	South Asian (n=32)	Black (n=22)	White (n=65)	p-value
Malar rash	65	33	65	66	36	78	<0.05
Arthritis	62	78	46	72	59	71	<0.05
Serorsitis	18	44	16	16	41	11	<0.05
Renal	36	33	51	28	59	22	<0.01
Immunologic	81	100	87	97	82	65	<0.001
dsDNA	66	56	75	81	73	49	<0.01
Anti-Sm	32	56	43	22	41	20	<0.05
aPL (ACL or LAC)	47	33	49	53	55	42	NS

Medications were prescribed equally across ethnicities; most patients were taking prednisone (75%), hydroxychloroquine (84%), and 56% required additional immunosuppression (azathioprine, methotrexate, mycophenolate mofetil or cyclophosphamide). Disease measures were similar across ethnicities, overall SLEDAI was 3.1 ± 4.2, SLAM 3.4 ± 3.7; SDI median 0.3 (range 0-5), and physician global VAS was 15 ± 23 (range 0-99).

## Conclusion

Canadian cSLE patients reflect our multi-ethnic population, with observed differences in disease manifestations, antibody profiles and health status by ethnicity.

## Disclosure

Deborah M. Levy: None; Earl D. Silverman: None; Lori B. Tucker: None; Gaelle Chédeville: None; Adam Huber: None; Janet E. Pope: None; Christine A. Peschken: None; CaNIOS Investigators: None.

